# Diagnostic Utility of Double-Echo Steady-State (DESS) MRI for Fracture and Bone Marrow Edema Detection in Adolescent Lumbar Spondylolysis

**DOI:** 10.3390/diagnostics13030461

**Published:** 2023-01-26

**Authors:** Atsushi Kitakado, Takeshi Fukuda, Jiro Kobayashi, Hiroya Ojiri

**Affiliations:** 1 Department of Radiology, Medical Scanning, 6-10-1 Nishi-Shinjuku-ku, Tokyo 160-0023, Japan; 2 Department of Radiology, The Jikei University School of Medicine, 3-25-8 Nishi-Shimbashi, Minato-ku, Tokyo 105-8461, Japan

**Keywords:** spondylolysis, pars interarticularis, fracture, bone marrow edema, magnetic resonance imaging, computed tomography

## Abstract

To evaluate the ability of double-echo steady-state (DESS) MRI to detect pars interarticularis fracture and bone marrow edema (BME) in spondylolysis, 500 lumber pars interarticularis from 50 consecutive patients (38 males and 12 females, mean age 14.2 ± 3.28 years) with spondylolysis who underwent both MRI and CT within 1 week were evaluated. All participants were young athletes who complained of lower back pain. Fractures were classified into four grades and CT was used as a reference; BME was evaluated in a binary manner and STIR was used as a reference. The diagnostic performance of fractures on DESS and T1WI, and BME on DESS was assessed by two radiologists independently. For fracture detection, DESS showed high diagnostic performance at a sensitivity of 94%, specificity of 99.5%, and accuracy of 98.8%, whereas T1WI showed lower sensitivity (70.1%). Fracture grading performed by DESS showed excellent agreement with CT grading (Kappa = 0.9). For BME, the sensitivity, specificity, and accuracy of DESS were 96.5%, 100%, and 99.6%, respectively. The inter-rater agreement of DESS for fracture and BME was 0.8 and 0.85, respectively. However, the inter-rater agreement for fracture on T1WI was 0.52. DESS had high diagnostic performance for fracture and BME in pars interarticularis. In conclusion, DESS had potential to detect all critical imaging findings in spondylolysis and may replace the role of CT.

## 1. Introduction

The reported prevalence of spondylolysis is high in 47% of athletes with back pain [1,2]. Although the cause of spondylolysis is not fully understood, it is thought to be a fracture at the pars interarticularis due to repetitive mechanical stress [3]. If spondylosis is left untreated, chronic nonunion may cause spondylolisthesis. Early detection and early medical intervention are the keys to avoiding such chronic complications [4].

Imaging plays a critical role in diagnosing spondylolysis because the sensitivity of physical examination is limited compared with imaging modalities [5]. Variable modalities have been used to detect spondylolysis, such as plain radiography, computed tomography (CT), bone scintigraphy, single-photon emission CT (SPECT), and magnetic resonance imaging (MRI). Plain radiography and CT have been used to detect pars interarticularis fracture. However, plain radiography has lower sensitivity than CT for fracture detection [6]. CT is regarded as the best modality to assess bony structure. Hence, morphological changes in pars interarticularis from fine cortical breaks to complete fracture at the late stage of spondylolysis are well delineated. However, in addition to the radiation exposure, CT has a disadvantage as a poor sensitivity in detecting symptom-related stress reaction, which occurs earlier than morphological changes [7]. Bone scintigraphy and SPECT can detect the metabolic abnormality prior to fracture appearance. However, increased metabolic activity is a nonspecific feature that can be seen in various diseases. Poor spatial resolution also limits its utility in spondylolysis. SPECT-CT can allow the localization of the precise anatomical sites of metabolic abnormality and is reported as a valuable modality [8]. However, ionizing radiation is a key drawback of SPECT-CT, and it has not been commonly used for first-line imaging modality in young athletes with back pain.

MRI is an alternative imaging modality without the risk of exposure of ionizing radiation. The sensitivity of MRI for fracture detection is less than CT, but it can detect bone marrow edema (BME) at the earlier phase of spondylolysis before a fracture occurs [8,9,10]. Historically, spondylolysis was classified into three stages as early, progressive, and terminal, based on the fracture status [11]. They have been correlated with the future fusion rate of the pars interarticularis fracture. Subsequently, MRI classification was proposed to classify pre-fracture spondylolysis [8]. According to this classification system, grade 1 is defined as the presence of BME localized in the pars interarticularis without fracture. Grade 2 and 3 are defined as presence of BME with incomplete and complete fracture at pars interarticularis, respectively. These stages accompanied with BME are sometimes named as acute spondylolysis. Hence, MRI is critical in diagnosing acute spondylolysis but has limited value in determining the fracture grade. On the other hand, grade 4 is a complete fracture without BME, and it may be called chronic spondylolysis [12]. In this stage, MRI is also useful to evaluate the late complication of spondylolysis, such as spondylolisthesis and spinal canal stenosis.

Nevertheless, due to its high diagnostic performance of CT in detecting fracture, CT still plays a critical role in accurate diagnosis, deciding the best clinical management, and predicting the outcome of spondylolysis. CT comes with radiation exposure for the vulnerable adolescent population that is usually experiencing growth spurts. Therefore, it would be substantially beneficial if MRI could adequately detect pars interarticularis fracture.

As double-echo steady state (DESS) reportedly visualizes the normal pars interarticularis better than T1-weighted imaging (T1WI) [13], DESS has been routinely used when spondylolysis was suspected in our institution. However, the diagnostic performance of DESS for spondylolysis has not been reported. Because our clinical experience suggested that DESS was better for detecting fractures than any other routine spine MRI protocols and its ability to detect BME seems high, we aimed to report the clinical utility of DESS in spondylolysis, which has rarely been documented in the literature.

In this study, we evaluated the ability of DESS to detect fractures in spondylolysis and determine whether DESS could potentially replace CT by retrospectively collecting young athletes with low back pain. In addition, we also evaluated whether DESS could detect BME at the pars interarticularis by using the short tau inversion recovery (STIR) sequence as a reference.

## 2. Materials and Methods

### 2.1. Subjects

From the medical record, we retrospectively recruited consecutive 50 patients with a confirmed diagnosis of spondylolysis who were scanned in our institution from December 2019 to February 2021. Included patients were young athletes who complained of recent onset or exacerbation of low back pain. The cases were excluded if the MRI and CT were not performed within a week. We also excluded cases without DESS sequences in the MRI protocol.

### 2.2. Scan Settings of MRI and CT

MRI was performed on 3T scanners (Magnetom Skyra, Siemens Healthineers, Erlangen, Germany) using 24-channel coils. In our institution, the protocol for patients who were suspected spondylolysis included the 3D DESS sequence (field of view: 300 × 220 mm, repetition time: 9.0 ms, echo time: 3.46 ms, slice thickness: 1 mm, flip angle: 20°, scan time: 2 min and 20 s), in addition to the standard protocols for spines. Standard protocols were sagittal T1WI, T2-weighted imaging (T2WI), and STIR with a slice thickness of 4.5 mm, and axial T1WI and T2WI with a slice thickness of 3.5 mm. DESS was reformatted in sagittal, oblique-sagittal, and oblique-axial orientations with a slice thickness of 1 mm. Oblique-sagittal orientation was defined as the section parallel to the long axis of the inferior articular process on the axial image. Oblique-axial images were obtained through the pars interarticularis.

CT was performed using a Somatom go Top (Siemens Healthineers, Forchheim, Germany) with the following parameters: 64 × 0.6 mm collimation, 0.4 mm; pixel spacing, 0.8 mm; pitch factor, CARE kV Quality ref.mAs@120 kV: 200 mAs; modulated tube current. Images were reconstructed in sagittal, oblique-sagittal, and oblique-axial orientations with a slice thickness of 1 mm. The oblique-sagittal and oblique-axial orientations were determined in the same manner as for DESS on MRI.

### 2.3. Imaging Analysis

To include the normal pars interarticularis as a control, we assessed the bilateral pars interarticularis of all lumber vertebras. All images were anonymized and independently analyzed in a randomized fashion by two board-certificated radiologists (A.K. and T.F.) with 16 and 12 years of experience, respectively.

**Figure 1:**
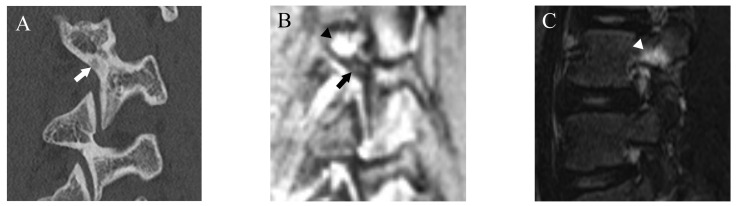
Representative images of grade 1 fracture. The right pars interarticularis of L3 from a 14-year-old male. (**A**) Oblique-sagittal CT image showing faint attenuation in the cortex (arrow). (**B**) The faint attenuation corresponds to slight hyperintensity in the cortex on an oblique-sagittal DESS image (arrow). The window of the image was adjusted to enhance the faint finding. Note the BME in the pedicle (arrowhead). (**C**) Sagittal STIR image shows BME in the right pedicle of L3 (arrowhead).

**Figure 2:**
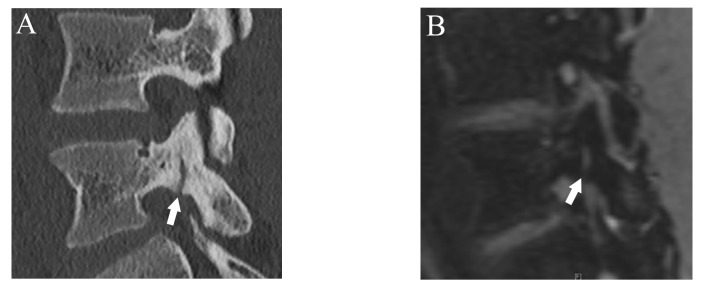
Representative images of grade 2 fracture. The left pars interarticularis of L5 from a 16-year-old male. (**A**) Sagittal CT image showing an incomplete but clear fracture line starting from the caudal part of the pars interarticularis (arrow). (**B**) Sagittal DESS image showing a linear high signal that suggests the fracture line (arrow).

**Figure 3:**
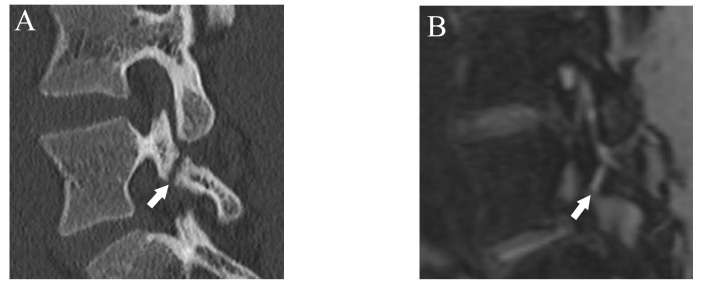
Representative images of grade 3 fracture. The right pars interarticularis of L5 from a 16-year-old male. (**A**) Sagittal CT image showing a complete fracture across the whole pars interarticularis (arrow). (**B**) Sagittal DESS image showing a linear high signal corresponding to the complete fracture (arrow).

Fractures were classified using a four-grade system in which 0 indicated no fracture, 1 indicated bone resorption, 2 indicated an incomplete fracture, and 3 indicated a complete fracture. Bone resorption (grade 1) was defined as faint bone resorption with an irregular or unclear margin in cortical bone (Figure 1). An incomplete or complete fracture was diagnosed when there was a clear linear attenuation in the typical part of the pars interarticularis (Figure 2 and Figure 3). Complete fracture (grade 3) was defined as a fracture through the pars interarticularis. If the fracture did not cross the whole pars interarticularis, it was graded as incomplete (grade 2). Sagittal, oblique-sagittal, and oblique-axial CT images were used to create standard reference for fracture. BME was graded as negative or positive, and sagittal STIR images were used to create a standard reference. If there is a high signal intensity at pars interarticularis on STIR, the case is considered as positive for BME. Standard references of fracture and BME were obtained by independent evaluation by two readers, and consensus results were created for statistical analysis. When there were disagreements between the two raters, consensus results were obtained through discussion.

After more than a 2-week interval from creating standard references, two readers independently analyzed fractures on T1WI and DESS using the same grading system as used in CT. On MRI, bone resorption (grade 1) can be detected as slight hyperintensity within cortical bone which normally shows homogeneous hypointensity (Figure 1). The fracture line on MRI is delineated as linear hypointensity on T1WI and linear hyperintensity on DESS (Figure 2 and Figure 3). DESS was also used to evaluate the BME binary. Similar to STIR, if there is a high signal at pars interarticularis on DESS, it is considered positive for BME. Consensus results for T1WI, DESS for fracture and DESS for BME were created for statistical analysis.

Additionally, the confidence of the interpretation was scored to evaluate the diagnostic confidence of T1WI and DESS for fracture and DESS for BME as either probable or definite.

Both raters were blinded to any clinical information and the images used for standard reference. The appearance order of T1WI and DESS images was randomized to avoid biased evaluation.

### 2.4. Statistical Analysis

For fracture assessment, the consensus CT results were used as the reference standard. After converting the fracture grade results into binary results with grade 0 indicating absence of fracture and grades 1–3 indicating presence of fracture, we calculated the sensitivity, specificity, and accuracy of T1WI and DESS for fracture detection. In addition, Cohen’s Kappa was used to see the accuracy of fracture grading by T1WI and DESS with using CT as a reference. For the BME assessment, the sensitivity, specificity, and accuracy of DESS were calculated with STIR as a reference. Again, to see the agreement for BME detection between DESS and STIR, we calculated Cohen’s Kappa.

To assess the diagnostic confidence, the frequencies of definite confidence in fracture assessment (T1WI, DESS) and BME assessment (DESS) were calculated for the two raters. The McNemer test was used to compare the diagnostic confidence between T1WI and DESS for fracture.

Cohen’s Kappa was used to calculate the inter-rater agreements for fracture and BME assessment. An interpretation of Kappa values was as follows. A value less than 0.21 was interpreted as slight agreement, 0.21–0.40 was interpreted as fair agreement, 0.41–0.60 was interpreted as moderate agreement, 0.61–0.80 was interpreted as substantial agreement, and 0.81 or greater was interpreted as excellent agreement.

A *p* value of less than 0.05 was considered statistically significant. All statistical analyses were performed with SPSS version 24.0 for Windows (IBM Japan, Tokyo, Japan).

## 3. Results

### 3.1. Subjects

**Figure 4:**
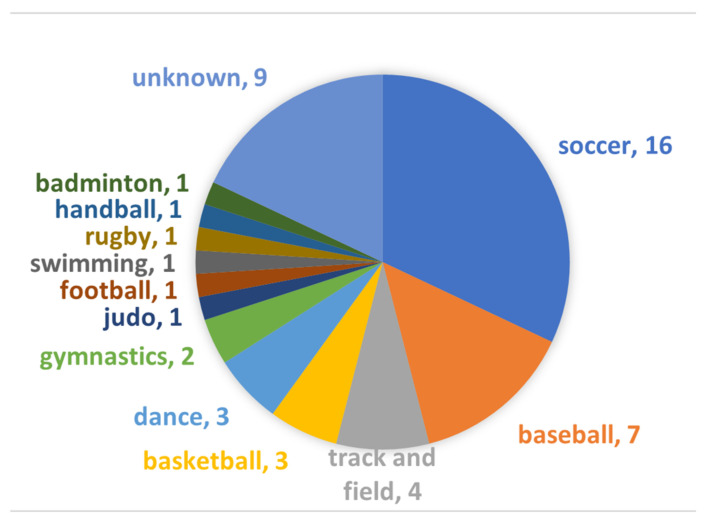
Breakdown of sports activities. The number indicates number of patients.

The cohort comprised 50 patients (38 males and 12 females, mean age 14.2 ± 3.28 years). None of them lacked DESS sequence, and all had the both MRI and CT within a week; thereby, all 50 subjects were included in this study. Participants were dedicated to variable sports (Figure 4).

The mean interval between MRI and CT was 3 ± 1.78 days. Out of 500 pars interarticularis, 67 fractured and 433 normal pars interarticularis were detected (Table 1). Fractures were detected from L3 to L5 level, and there were no patients who had the lesion at L1 or L2.

Overall, 40 of 50 patients showed BME for at least one pars interarticularis, whereas 10 patients did not have BME.

### 3.2. Fracture Assessment

**Figure 5:**
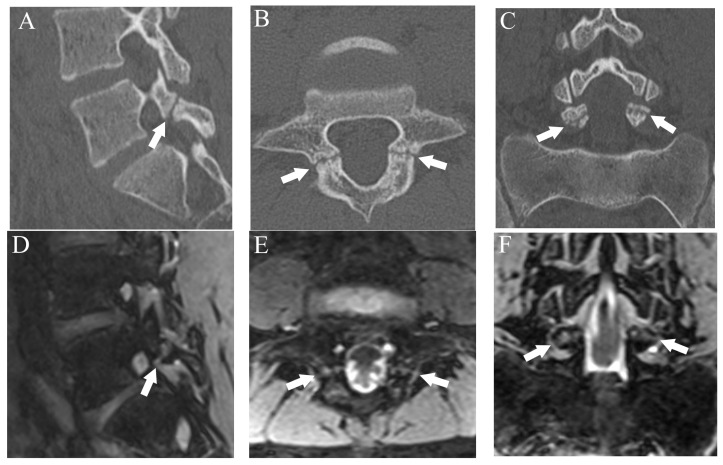
Comparison of bilateral grade 3 fractures at L5 from an 11-year-old male in multiple planes between CT and DESS. (**A**–**C**) CT shows complete fractures in L5 pars interarticularis on the right-sided sagittal, axial, and coronal plane, respectively (arrows). (**D**–**F**) DESS shows comparable complete fracture on the right-sided sagittal, axial, and coronal plane, respectively (arrows).

**Figure 6:**
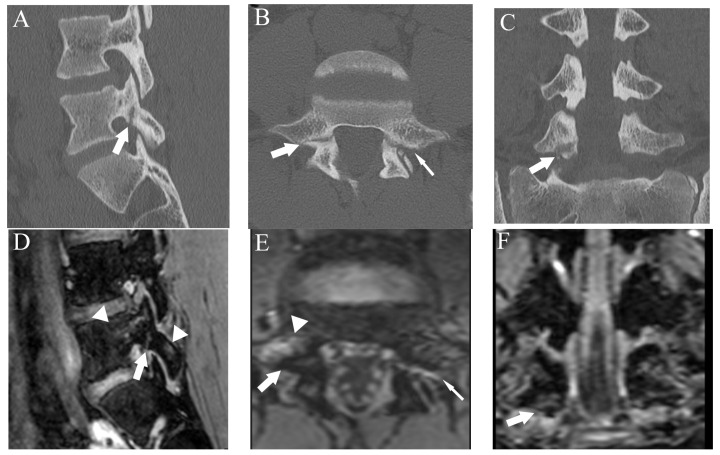
Comparison of grade 2 fracture at the right pars interarticularis at L5 from a 14-year-old male in multiple planes between CT and DESS. (**A**–**C**) CT shows grade 2 fracture on the right pars interarticularis on the right-sided sagittal, axial, and coronal plane, respectively (arrows). On the axial plane, fracture of the left side is also delineated (thin arrow). (**D**–**F**) DESS shows grade 2 fractures comparable to CT on the right-sided sagittal, axial, and coronal plane, respectively (arrows). The sagittal and axial images delineate BME around the fracture (arrowhead), and the left pars interarticularis fracture is also delineated on the axial image (thin arrow).

Regarding the fracture detection using CT as a reference, the respective sensitivity, specificity, and accuracy were 70.1%, 100%, and 96% for T1WI and 94%, 99.5%, and 98.8% for DESS (Figure 5 and Figure 6).

**Figure 7:**
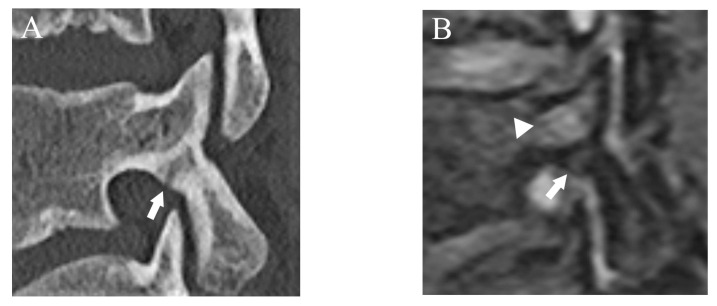
The case that lower fracture grade was assigned by DESS than CT. (**A**) CT suggests an incomplete fracture which was consistent with grade 2 (arrow). (**B**) DESS shows a subtle high signal in the cortex of the pars interarticularis and evaluated as grade 1 (arrow). BME is also detected (arrowhead).

Agreement of fracture grading with CT showed excellent (Kappa = 0.90 (95% confidence interval (CI): 0.84, 0.96)) on DESS but T1WI showed substantial (Kappa = 0.67 (95% CI: 0.61, 0.73)) agreement. Compared to reference fracture grade created by CT, nine cases were assigned a lower grade by DESS, whereas three were assigned a higher grade (Figure 7).

### 3.3. BME Assessment

The sensitivity, specificity, and accuracy of DESS for BME were 96.5%, 100%, and 99.6%, respectively. The Kappa value for BME detection between DESS and STIR was 0.98 (95% CI: 0.71, 1.00), which means excellent agreement.

### 3.4. Diagnostic Confidence

Compared with T1WI, DESS showed a significantly higher prevalence of definite confidence for fracture assessment by both raters (25.5% vs. 88.3%, *p* < 0.01 for rater 1; 37.6% vs. 96.6%, *p* < 0.01 for rater 2). There was also a high prevalence of definite confidence in evaluating BME with DESS (96.1% for rater 1 and 97.2% for rater 2).

### 3.5. Inter-Rater Agreement

The items with a high Kappa statistic of 0.6 or greater were fracture on CT (0.80 (95% confidence interval (CI): 0.74, 0.86)), fracture on DESS (0.80 (95% CI: 0.74, 0. 86)), BME on STIR (0.86 (95% CI: 0.77, 0.95)), and BME on DESS (0.85 (95% CI: 0.76, 0.93)). However, the inter-rater agreement for fracture on T1WI was moderate (0.52 (95% CI: 0.45, 0.58)).

## 4. Discussion

The present study showed that the ability of DESS to detect pars interarticularis fracture is comparable to CT, and DESS may replace CT for fracture assessment in spondylolysis. In addition, the ability of DESS to detect BME was also comparable to STIR, which means that all essential imaging findings related to spondylolysis may be evaluated using only DESS regardless of spondylolysis stages as early, progressive, and terminal. However, it would be more realistic to add DESS rather than replace the conventional MRI sequences because differential diagnoses for low back pain in young athletes are variable.

Conventionally, the diagnosis of lumbar spondylolysis has been made by combining BME assessment on MRI and fracture assessment on CT. CT reportedly has higher sensitivity and specificity for fractures than TIWI [14]. Therefore, CT is still used to assess the young population who are sensitive to radiation. Nuclear imaging, especially SPECT, shows increased tracer uptake for bone stress response even before a fracture occurs [15,16,17]. SPECT was used as a gold standard for spondylolysis in several imaging studies to see the diagnostic performance of other imaging modalities [5,18,19]. However, tracer uptake is a non-specific phenomenon, and precise localization of metabolic abnormality is difficult. In addition, abnormal uptake is absent in terminal spondylolysis, and due to its radiation exposure, nuclear imaging is currently not frequently used for spondylolysis in clinical practice [20].

DESS is a three-dimensional (3D) coherent steady-state gradient-echo sequence used mainly for articular cartilage evaluation [21]. The high spatial resolution of DESS is also reportedly valuable in other musculoskeletal areas, such as assessing the extrinsic ligament of the wrist [22]. Recently, 3D DESS was reported to point out more symptomatic nerve compression due to disc herniation than conventional MRI sequences [23]. It is difficult to observe all potential sites for a herniated disc to compress the nerve with conventional MRI sequences, which is mainly due to a limited number of acquired slices per intervertebral disc. Especially, conventional sequences may fail to detect disc herniation outside the intervertebral foramen. However, DESS could delineate nerve roots and their relationship with a herniated disc better than conventional sequences. Thereby, DESS had additional value in the assessment of intervertebral disc herniation. Campbell et al. compared the visibility of the pars interarticularis in healthy volunteers using T1WI and DESS [13]. They implied that 3D DESS could be a suitable method to evaluate fracture of the pars interarticularis based on the higher inter-rater agreement for reconstructed DESS than T1WI for normal pars interarticularis visualization. However, at that time, they concluded that the 3D sequence was too time-consuming to be used routinely. This problem is almost resolved with the recently developed 3T scanner, enabling our 3D DESS images to be obtained within 3 min.

The fracture line was showed on DESS as a high signal gap in the pars interarticularis, which is consistent with a low attenuation fracture line on CT. DESS showed a comparable fracture detection ability to CT and enabled sufficient classification of the fracture pattern. Bone resorption, which we defined as faint bone resorption with an irregular or unclear margin, is clinically significant because it is considered a very early stage of fracture and its detection may improve the management and clinical prognosis [5,24]. In the present study, there were 12 bone resorption cases, and only two of them were correctly diagnosed by T1WI. However, DESS could detect nine of them. When we carefully assessed the signal change at the caudal cortex of the pars interarticularis with DESS, bone resorption cases showed a faint high signal with unclear boundaries and were confirmed to have bone resorption on CT. Although the diagnostic performance of T1WI for fracture was not poor due to a large number of normal pars interarticularis in our cohort, diagnostic confidence by both raters was significantly lower than DESS. Hence, both raters considered T1WI, our routine protocol, was not suitable to visualize fractures at the pars interarticularis properly. The inter-rater agreement in diagnosing fracture was also moderate on T1WI, whereas it was almost excellent on DESS. Based on the above-mentioned findings, DESS may be able to replace the routinely used 2D T1WI for fracture assessment.

Ang et al. assessed the detectability of fracture at the pars interarticularis by 3D T1 volume interpolated breath-hold examination (VIBE) with CT as a reference [25]. In their cohort of 24 patients, the overall sensitivity and specificity of 3D T1 VIBE for fracture detection were 97.7% and 92.3%, respectively, which is comparable to our results with DESS. Our study may have more clinical value than the study by Ang et al. because we evaluated bone resorption as a distinct pattern of fracture and had a larger sample size. In the previous study of 3D T1 VIBE, false-positive and false-negative results were reported for cases in which the vertebral facet showed marked hypertrophic sclerosis [25]. We did not find any cases with a marked sclerotic facet, but there is still a discrepancy in fracture delineation between DESS and CT. For example, a subtle thin fracture on CT failed to be detected on DESS. We thought that the raters missed the fracture on DESS due to the absence of a high signal at the fracture site. One potential explanation for this is that decreased inflammatory water contents at the fracture site may preclude the increase in the signal on DESS during the early phase of fracture healing before the fracture line disappears on CT.

BME is also a critical imaging finding in spondylolysis that is thought to reflect stress-related pars interarticularis injury due to repetitive trauma and appears before the fracture becomes visible on CT [8,10]. Therefore, the ability of DESS to detect BME adds to its clinical value, which surpasses 2D or 3D T1WI. In addition, BME could be a helpful finding to improve the detectability of subtle fractures because raters would more carefully evaluate the presence of fractures in cases with BME. Generally, gradient-recalled echo sequences such as DESS are thought to be insensitive to bone marrow abnormalities due to the magnetic susceptibility of trabecular bone. DESS was previously reported to be less sensitive in detecting BME than the fat-suppressed spin echo sequence [26]. However, our results showed that DESS had high sensitivity and specificity for detecting BME compared with STIR. This may be because our study population might have had relatively strong BME, and the BME signal might have surpassed the magnetic susceptibility of trabecular bone.

The present study had several limitations. First, although we included a relatively large number of subjects compared with previous spondylolysis studies related to imaging [14,24,25,27], a prospective study with a larger sample size is warranted. Second, we did not include the healthy control because DESS was only used for spondylolysis cases in our institution. However, we evaluated all lumber vertebras to include the control finding. We believe whole lumber vertebral assessment and evaluation by two blinded independent readers could reduce the bias. Third, we did not assess the visibility of the healing process on follow-up images. Hence, we are planning to see and investigate the follow-up cases in the future. Forth, we did not create oblique-sagittal and oblique-axial images for T1WI because we did not use 3D T1WI as a routine protocol, and our primary purpose was to see the diagnostic performance of DESS for fracture and BME. It would be interesting to compare DESS with currently available CT-like imaging, such as fast field echo resembling a CT using restricted echo-spacing (FRACTURE) [28,29]. Furthermore, it would be important to assess the utility of DESS for spondylolysis with spine surgeons through surgical cases in the future.

## 5. Conclusions

DESS had a high diagnostic performance for fracture detection and fracture grading of pars interarticularis in spondylolysis. It also had a similar ability to detect BME with STIR. Hence, DESS had the potential to detect all critical imaging findings in spondylolysis and may add to the role of MRI. The use of DESS rather than CT would be substantially beneficial for young populations who are susceptible to spondylolysis and are sensitive to radiation exposure.

## Figures and Tables

**Table 1:** Number of pars interarticularis for each fracture grade.

Grade	Definition	Number
0	Normal	433 (86.6)
1	Bone resorption	12 (2.4)
2	Incomplete fracture	24 (4.8)
3	Complete fracture	31 (6.2)

The number in parenthesis is percentage.

## Data Availability

The data presented in this study are available on request from the corresponding author.
